# Histone deacetylase 8 inhibition prevents the progression of peritoneal fibrosis by counteracting the epithelial-mesenchymal transition and blockade of M2 macrophage polarization

**DOI:** 10.3389/fimmu.2023.1137332

**Published:** 2023-02-23

**Authors:** Xun Zhou, Hui Chen, Yingfeng Shi, Jinqing Li, Xiaoyan Ma, Lin Du, Yan Hu, Min Tao, Qin Zhong, Danying Yan, Shougang Zhuang, Na Liu

**Affiliations:** ^1^ Department of Nephrology, Shanghai East Hospital, Tongji University School of Medicine, Shanghai, China; ^2^ Department of Medicine, Rhode Island Hospital and Alpert Medical School, Brown University, Providence, RI, United States

**Keywords:** histone deacetylase 8, epithelial-mesenchymal transition, epidermal growth factor receptor, macrophage polarization, peritoneal fibrosis

## Abstract

**Background:**

Peritoneal dialysis (PD) is an effective replacement therapy for end-stage renal disease patients. However, long-term exposure to peritoneal dialysate will lead to the development of peritoneal fibrosis. Epigenetics has been shown to play an important role in peritoneal fibrosis, but the role of histone deacetylases 8 (HDAC8) in peritoneal fibrosis have not been elucidated. In this research, we focused on the role and mechanisms of HDAC8 in peritoneal fibrosis and discussed the mechanisms involved.

**Methods:**

We examined the expression of HDAC8 in the peritoneum and dialysis effluent of continuous PD patients. Then we assessed the role and mechanism of HDAC8 in peritoneal fibrosis progression in mouse model of peritoneal fibrosis induced by high glucose peritoneal dialysis fluid by using PCI-34051. In vitro, TGF-β1 or IL-4 were used to stimulate human peritoneal mesothelial cells (HPMCs) or RAW264.7 cells to establish two cell injury models to further explore the role and mechanism of HDAC8 in epithelial-mesenchymal transition (EMT) and macrophage polarization.

**Results:**

We found that HDAC8 expressed highly in the peritoneum from patients with PD-related peritonitis. We further revealed that the level of HDAC8 in the dialysate increased over time, and HDAC8 was positively correlated with TGF-β1 and vascular endothelial growth factor (VEGF), and negatively correlated with cancer antigen 125. In mouse model of peritoneal fibrosis induced by high glucose dialysate, administration of PCI-34051 (a selective HDAC8 inhibitor) significantly prevented the progression of peritoneal fibrosis. Treatment with PCI-34051 blocked the phosphorylation of epidermal growth factor receptor (EGFR) and the activation of its downstream signaling pathways ERK1/2 and STAT3/HIF-1α. Inhibition of HDAC8 also reduced apoptosis. In vitro, HDAC8 silencing with PCI-34051 or siRNA inhibited TGF-β1-induced EMT and apoptosis in HPMCs. In addition, continuous high glucose dialysate or IL-4 stimulation induced M2 macrophage polarization. Blockade of HDAC8 reduced M2 macrophage polarization by inhibiting the activation of STAT6 and PI3K/Akt signaling pathways.

**Conclusions:**

We demonstrated that HDAC8 promoted the EMT of HPMCs via EGFR/ERK1/2/STAT3/HIF-1α, induced M2 macrophage polarization via STAT6 and PI3K/Akt signaling pathways, and ultimately accelerated the process of peritoneal fibrosis.

## Introduction

Peritoneal dialysis (PD) is an effective renal replacement therapy (1), however, the proportion of ESRD patients treated with PD is lower than hemodialysis in developed countries (2). Clinical studies have demonstrated that peritoneal ultrafiltration declines gradually 2 - 4 years after the initiation of PD (3–5). The induction of peritoneal fibrosis is a complex pathological event, and is characterized by epithelial-to-mesenchymal transition (EMT), activation of fibroblasts, deposition of extracellular matrix (ECM) components and angiogenesis (6). The release of several growth factors/cytokines, especially transforming growth factor-1 (TGF-β1) and vascular endothelial growth factor (VEGF), and activation of various signaling pathways contribute to the progression of peritoneal fibrosis. Our previous studies have demonstrated that epidermal growth factor receptor (EGFR) promotes the development and progression of peritoneal fibrosis *via* the activation of multiple pro-fibrosis signaling pathways, inflammatory responses and angiogenesis (7). The phosphorylation of EGFR subsequently leads to the activation of several intracellular signaling pathways, including extracellular signal-regulated kinases 1/2 (ERK 1/2) and signal transducer and activator of transcription 3 (STAT3) during peritoneal fibrosis (8). Thus, targeting EGFR may be an effective approach to preserve peritoneal membrane ultrafiltration capacity.

Macrophages are key components of the peritoneal immune system (9). Macrophages are usually divided into two functional subtypes (M1 and M2), which play different roles in various physiological and pathological environments (10). Analysis of experimental evidences from PD patient samples demonstrates that the majority of peritoneal macrophages tend to be M2 macrophages in phenotype and function (11). Predominance of M2 macrophage response leads to induction of EMT, and upregulate the production of ECM protein, angiogenesis, and fibrosis (12). Our previous studies have demonstrated that M2 macrophage polarization in the peritoneum is regulated by the phosphorylation of signal transducer and activator of transcription 6 (STAT6) and phosphatidylinositol-3-kinase (PI3K)/Akt pathways (13).

According to previous studies by our group, epigenetic regulation plays an important role in the process of peritoneal fibrosis (6, 7, 13, 14). Histone deacetylase 8 (HDAC8) is a class I HDAC that catalyzes the deacetylation of histones and non-histones (15). It has been demonstrated that HDAC8 plays a multifunctional role in cancer progression, such as stimulating tumor growth and metastasis by enhancing cell proliferation and activating EMT *via* acting on histones and non-histone substrates (15). However, the specific mechanism and target proteins of HDAC8 in regulating peritoneal fibrosis have not been revealed.

In this research, we examined the expression of HDAC8 in the peritoneum and dialysis effluent of continuous PD patients. Then we assessed the role and mechanism of HDAC8 in peritoneal fibrosis progression in mouse model of peritoneal fibrosis induced by high glucose peritoneal dialysis fluid (PDF) by using PCI-34051. *In vitro*, TGF-β1 and IL-4 were used to stimulate human peritoneal mesothelial cells (HPMCs) and RAW264.7 cells to establish two cell injury models to further explore the role and mechanism of HDAC8 in EMT and macrophage polarization.

## Materials and methods

Additional details for all methods are provided in the **Supplementary Material and Methods**.

### Clinical sample collection and ethics statement

We collected peritoneal tissue during catheterization initiation operations (n = 6) and refractory peritonitis-induced catheter migration (n = 6) at Shanghai East Hospital affiliated with Tongji University and completed co-immunofluorescent staining of HDAC8 and α-SMA. Human PD effluents from patients with different durations were collected at Shanghai East Hospital, Shanghai Baoshan Hospital and Shanghai Songjiang District Central Hospital from September 2017 to March 2022. These patients were divided into 5 groups according to duration: group 1 (duration ≤ 1 month, n=16), group 2 (1<duration ≤ 12 months, n=22), group 3 (12<duration ≤ 24 months, n=20), group 4 (24<duration ≤ 36 months, n=16), and group 5 (duration>36 months, n=14). This study was approved by the Medical Ethics Committee of Shanghai East Hospital and was conducted in accordance with the Declaration of Helsinki. Written informed consent was obtained from each patient. And we have obtained the registration number from the Chinese Clinical Trial Register (ChiCTR): ChiCTR2100052103.

### Animals and treatment

Male C57BL mice (purchased from Shanghai Super-B&K Laboratory Animal Corp. Ltd) weighed 20-25g were used in this study. The peritoneal fibrosis model was created by daily i.p. injection of 100 ml/kg peritoneal dialysis fluid with 4.25% glucose for 28 days (6). To examine the efficacy of PCI-34051 in peritoneal fibrosis, two different concentrations of PCI-34051 (10 or 20 mg/kg) in DMSO was intraperitoneally every day and the mice were sacrificed on day 28 to collect peritoneum. The animal protocol was reviewed and approved by the Institutional Animal Care and Use Committee at Tongji University (Shanghai, China). The details of animals and treatment are provided in the **Supplementary Material and Methods**.

### Cell culture and treatments

Cells were collected and cultured as described previously (13, 16). HPMCs (American Type Culture Collection, ATCC; Rockville, MD, United States) were cultured in Dulbecco’s modified Eagle’s medium (DMEM) with F12 containing 10% fetal bovine serum (FBS), 1% penicillin, and streptomycin. Raw264.7 cells (American Type Culture Collection, ATCC; Rockville, MD, United States) were cultured in RPMI-1640 medium containing 10% FBS, 1% penicillin and streptomycin. All cells were cultured in an atmosphere of 5% CO2, and 95% air at 37°C and were experimented after three generations. To examine the role and mechanisms of HDAC8 in TGF-β1-induced EMT of HPMCs, we starved HPMCs for 24 hours with DMEM/F12 containing 0.5% FBS and then exposed to TGF-β1 (2ng/ml) in the presence or absence of PCI-34051 (5mM). for 36 hours. To examine the role and mechanisms of HDAC8 in IL-4-induced macrophage polarization of Raw264.7 cells, we starved Raw264.7 cells for 24 hours with RPMI-1640 containing 0.5% FBS and then exposed to IL-4 (10 ng/ml) for 24 hours in the presence or absence of PCI-34051 (5μM). Then, cells were harvested for further immunoblot analysis or immunofluorescent staining. All the *in vitro* experiments were repeated at least three times.

## Results

### HDAC8 is highly expressed in peritoneum from long-term PD patients, positively correlated with TGF-β1 and VEGF and negatively correlated with CA125 in human PD effluent

**Figure 1:**
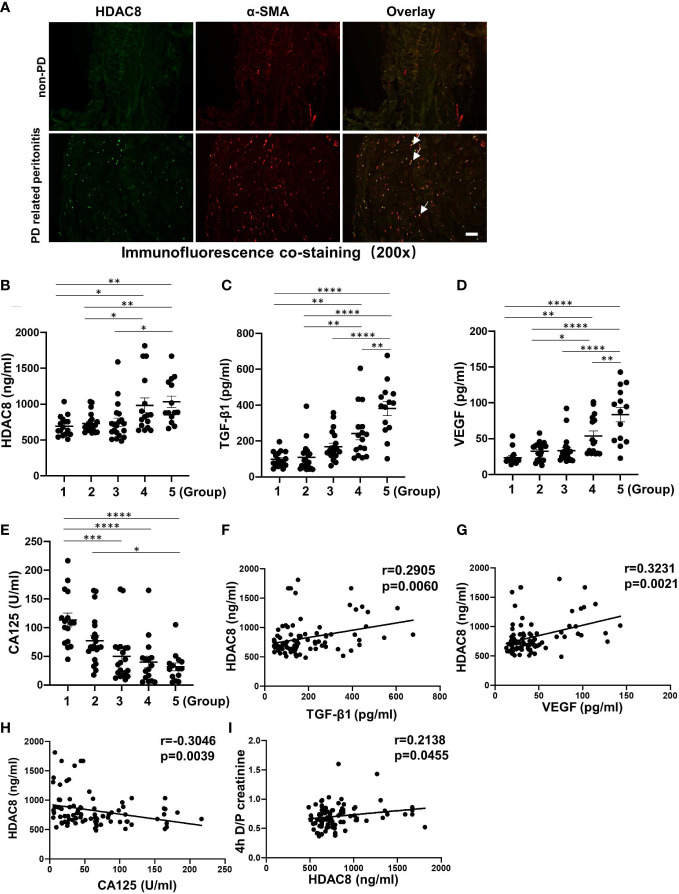
HDAC8 is highly expressed in peritoneum from long-term PD patients, positively correlated with TGF-β1 and VEGF and negatively correlated with CA125 in human PD effluent **(A)** Immunofluorescent co-staining of α-SMA and HDAC8 in the peritoneum from patients with non-PD and PD-related peritonitis. And HDAC8 was co-expressed with α-SMA-positive cells (white arrows). **(B–E)** Levels of HDAC8 and cytokines in dialysis effluent according to ELISA kits. The dialysis effluents from 88 PD patients were divided into 5 groups according to duration: group 1 (duration ≤ 1 month, n=16), group 2 (1<duration ≤ 12 months, n=22), group 3 (12<duration ≤ 24 months, n=20), group 4 (24<duration ≤ 36 months, n=16), and group 5 (duration>36 months, n=14). The expression levels of HDAC8 **(B)**, TGF-β1 **(C)**, VEGF **(D)**, and CA125 **(E)** were indicated in each group. **(F–I)** HDAC8 was positively correlated with enhanced expression of TGF-β1, VEGF and 4h D/P creatinine, and negatively with CA125 in dialysis effluent of PD patients. Correlation analysis were conducted between HDAC8 and TGF-β1 **(F)**, HDAC8 and VEGF **(G)**, HDAC8 and CA125 **(H)**, HDAC8 and 4h D/P creatinine **(I)**. Data were expressed as means ± SEM. *P<0.05; **P<0.01; ***P<0.001; ****P<0.0001. N.S., statistically not significant, with the comparisons labeled. All scale bars = 50 μm.

We collected peritoneal tissue during catheterization initiation operations (n = 6) and refractory peritonitis-induced catheter migration (n = 6) and completed co-immunofluorescent staining of HDAC8 and α-SMA. As shown in **Figure 1A**, HDAC8 was highly expressed in the thickened peritoneum of long-term PD patients with PD-related peritonitis, and HDAC8 was co-expressed with α-SMA positive cells.

ELISA kit assays showed that the expressions of HDAC8, TGF-β1 and VEGF in dialysis effluent were significantly increased, while the level of CA125 decreased with the prolongation of dialysis time (**Figures 1B–E**). CA125 is reported to be a marker of peritoneal mesothelial cell, with the severity of peritoneal fibrosis, the expression of CA125 is decreased due to the loss of peritoneal mesothelial cell (17). Further correlation analysis showed that HDAC8 was positively correlated with TGF-β1 (r = 0.2905, p = 0.0060) and VEGF (r= 0.3231, p = 0.0021), and negatively correlated with CA125 (r= −0.3046, p = 0.0039) (**Figures 1F–H**). In addition, it has been reported that patients characterized as high transporters had an increased sub-mesothelial fibrous layer (18). According to the results of PET tests in PD patients (**Figure 1I**), HDAC8 was positively correlated with the peritoneal transport rate of patients (r= 0.2138, p = 0.0455). **Table S1** showed the clinical characteristics of the enrolled PD patients. There was no significant difference between different groups of PD patients except creatinine and serum sodium. These data suggest that HDAC8 might be a clinically noninvasive biomarker in dialysis efflux for predicting peritoneal injury and transport status in PD patients.

### Inhibition of HDAC8 suppresses development of peritoneal fibrosis and improves functional impairments of peritoneal membrane in the high glucose PDF-injured peritoneum

**Figure 2:**
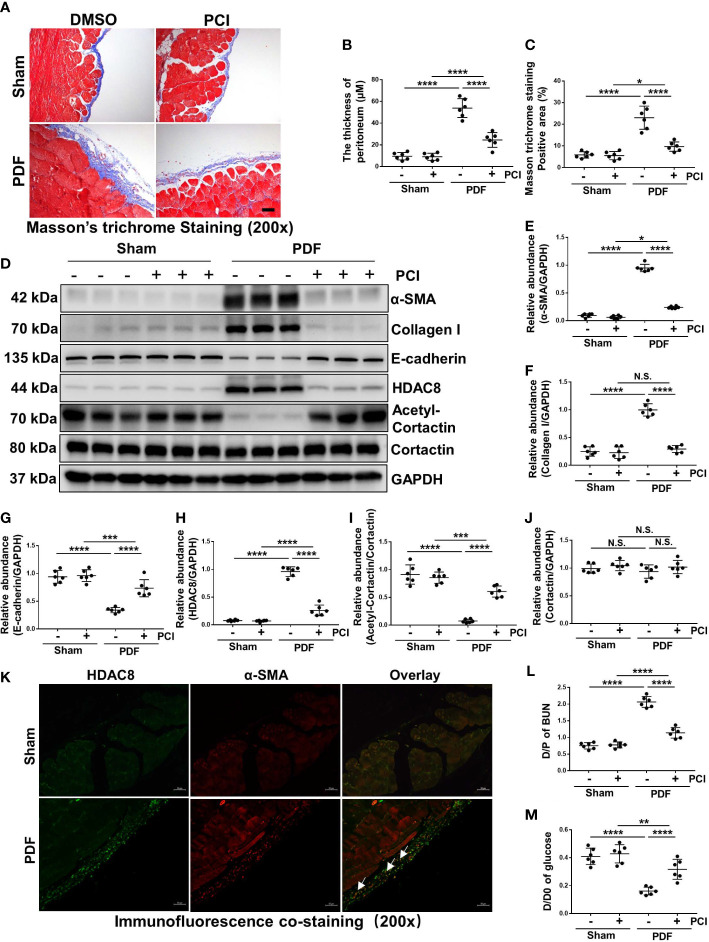
Inhibition of HDAC8 suppresses development of peritoneal fibrosis and improves functional impairments of peritoneal membrane in the high glucose PDF-injured peritoneum **(A)** Masson trichrome staining of the peritoneum in different groups of mice. **(B)** The thickness of peritoneum according to Masson trichrome staining. **(C)** The positive area of Masson trichrome staining-positive sub-mesothelial area (blue). **(D)** Western blot analysis showed the protein levels of α-SMA, collagen I, E-cadherin, HDAC8, cortactin, acetyl-cortactin and GAPDH in peritoneum from different groups of mice. Expression levels of **(E)** α-SMA, **(F)** collagen I, **(G)** E-cadherin, **(H)** HDAC8, **(I)** acetyl-cortactin, **(J)** cortactin in different groups were quantified by densitometry and normalized with GAPDH and cortactin respectively. **(K)** Immunofluorescent co-staining of α-SMA and HDAC8 (white arrows) in the peritoneum from mice with or without high glucose PDF injection. **(L)** The dialysate-to-plasma (D/P) ratio of blood urea nitrogen (BUN). **(M)** Ratio of dialysate glucose at 2 h after PDF injection to dialysate glucose at 0 hour (D/D0). Data were expressed as means ± SEM. *P<0.05; **P<0.01; ***P<0.001; ****P<0.0001. N.S., statistically not significant, with the comparisons labeled. All scale bars = 50 μm.

In the preliminary experiment, we used two different concentrations of PCI-34051 (10mg/kg or 20mg/kg) to intervene high glucose PDF induced peritoneal fibrosis in mice. Our results showed that two different concentrations of PCI-34051 could prevent the development of peritoneal fibrosis and improve functional impairments of peritoneal membrane in the high glucose PDF-injured peritoneum, and high concentration of PCI (20mg/kg) had a better effect on inhibiting peritoneal fibrosis and conserving peritoneum function (**Figure S1**). Therefore, we chose a concentration of 20mg/kg PCI-34051 to treat mice for further study. As shown by Masson trichrome staining in **Figure 2A**, we successfully established a mouse model of peritoneal fibrosis with continuous intraperitoneal injection of 4.25% glucose PDF, which was characterized by an increase in the thickness of the sub-mesothelial area. Treatment of PCI-34051 (20mg/kg/d) after injection of 4.25% glucose PDF significantly reduced these pathological changes (**Figures 2A–C**), suggesting that HDAC8 is a key mediator of peritoneal fibrosis. EMT is an important mechanism of peritoneal fibrosis (19, 20). We examined the expression of α-SMA, collagen I and E-cadherin by western blot. As shown in **Figures 2D–G**, expressions of α-SMA and collagen I were significantly increased, and expression of E-cadherin was decreased after exposure to 4.25% glucose PDF. Treatment of PCI-34051 blocked the EMT process.

To demonstrate the effectiveness of PCI-34051 *in vivo*, we examined the impact of PCI-34051 on the expression of HDAC8 and its substrate protein cortactin. The expression of HDAC8 increased significantly after the injection of 4.25% glucose PDF for 28 days, while the expression of acetyl-cortactin decreased (**Figures 2D, H–J**). Treatment of PCI-34051 decreased the expression of HDAC8 to the base level in mice receiving 4.25% glucose PDF, and increased acetyl-cortactin. Immunofluorescent staining showed that HDAC8 was mainly expressed in cells present in the sub-mesothelial zone and co-localized with α-SMA (**Figure 2K**). These results suggested that PCI-34051 may prevent the progression of peritoneal fibrosis by suppressing EMT. In addition, PCI-34051 improved high glucose PDF-associated peritoneal functional impairments according to the PET test results (**Figures 2L, M**).

### Inhibition of HDAC8 blocks the activation of EGFR/ERK1/2/STAT3/HIF-1α signaling pathway in the peritoneum exposed to high glucose dialysate

**Figure 3:**
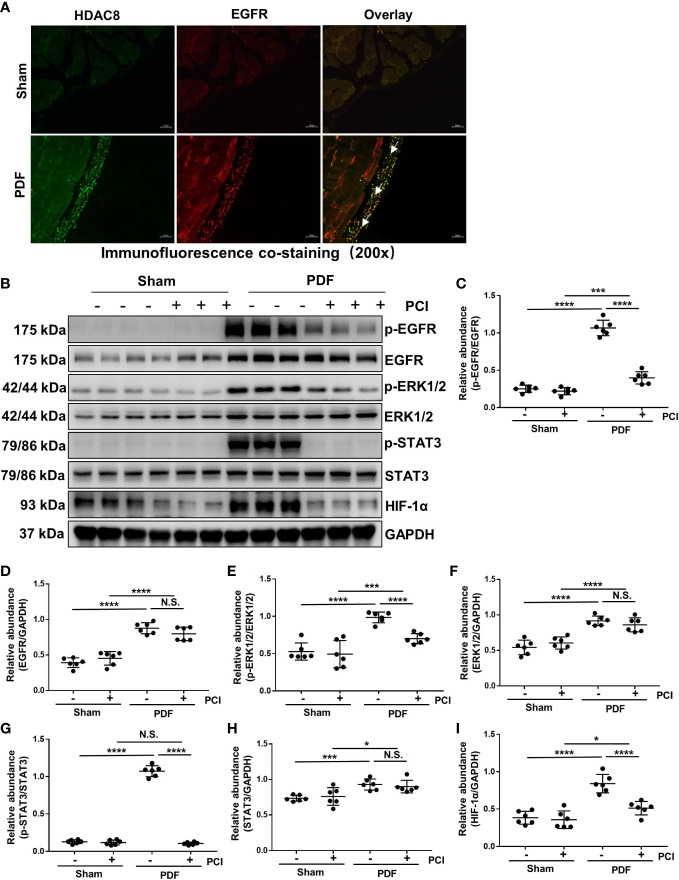
Inhibition of HDAC8 blocks the activation of EGFR/ERK1/2/STAT3/HIF-1α signaling pathway in the peritoneum exposed to high glucose dialysate **(A)** Immunofluorescent co-staining of EGFR and HDAC8 (white arrows) in the peritoneum from mice with or without high glucose PDF injection. **(B)** Western blot analysis showed the protein levels of p-EGFR, EGFR, p-ERK1/2, ERK1/2, p-STAT3, STAT3, HIF-1α and GAPDH. Expression levels of **(C)** p-EGFR, **(D)** EGFR, **(E)** p-ERK1/2, **(F)** ERK1/2, **(G)** p-STAT3, **(H)** STAT3, **(I)** HIF-1α were quantified by densitometry and normalized with GAPDH, EGFR, ERK1/2 and STAT3 respectively. Data were expressed as means ± SEM. *P<0.05; **P<0.01; ***P<0.001; ****P<0.0001. N.S., statistically not significant, with the comparisons labeled. All scale bars = 50 μm.

Studies from our group have demonstrated that the activation of EGFR/ERK/1/2/STAT3 signaling pathway is related to the progression of peritoneal fibrosis (21, 22). Therefore, we investigated the effect of HDAC8 on the activation of EGFR and its downstream signaling molecules ERK1/2 and STAT3. Immunofluorescent staining showed that HDAC8 was observed in EGFR positive cells (**Figure 3A**). As shown in **Figures 3B–H**, the phosphorylation of EGFR, ERK1/2 and STAT3 were significantly increased after exposure to 4.25% glucose PDF, while PCI-34051 administration inhibited their phosphorylation. Recent studies have demonstrated that the expression of HIF-1α in mesenchymal cells is mainly dependent on the activation of STAT3, and the inhibition of STAT3 will further suppressed the activation of HIF-1α, thus affecting the EMT of mesothelial cells (23). As shown in **Figures 3B, I**, the expression of HIF-1α was significantly increased after exposure to 4.25% glucose PDF, while PCI-34051 administration inhibited its expression. These data suggested that inhibition of HDAC8 suppressed the activation of the EGFR/ERK1/2/STAT3/HIF-1α signaling pathway.

### Blockade of HDAC8 with PCI-34051 or siRNA abrogates EMT by inhibition of EGFR/ERK1/2/STAT3/HIF-1α signaling pathway

**Figure 4:**
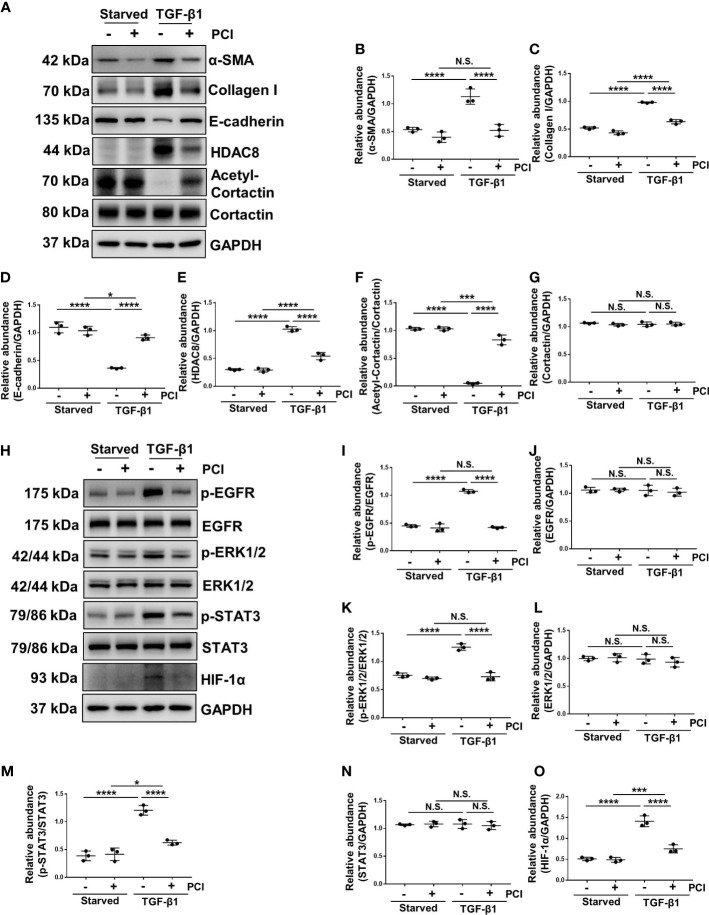
Blockade of HDAC8 with PCI-34051 abrogates EMT by inhibition of EGFR/ERK1/2/STAT3/HIF-1α signaling pathway **(A)** Serum-starved HPMCs were pretreated with PCI-34051 (5μM) and then exposed to TGF-β1 (2 ng/ml) for 36 h. Cell lysates were subjected to immunoblot analysis with specific antibodies against α-SMA, collagen I, E-cadherin, HDAC8, cortactin, acetyl-cortactin and GAPDH. Expression levels of **(B)** α-SMA, **(C)** collagen I, **(D)** E-cadherin, **(E)** HDAC8, **(F)** acetyl-cortactin, **(G)** cortactin in different groups were quantified by densitometry and normalized with GAPDH and cortactin respectively. **(H)** Cell lysates were subjected to immunoblot analysis with specific antibodies against p-EGFR, EGFR, p-ERK1/2, ERK1/2, p-STAT3, STAT3, HIF-1α and GAPDH. Expression levels of **(I)** p-EGFR, **(J)** EGFR, **(K)** p-ERK1/2, **(L)** ERK1/2, **(M)** p-STAT3, **(N)** STAT3, **(O)** HIF-1α were quantified by densitometry and normalized with GAPDH, EGFR, ERK1/2 and STAT3 respectively. Data were expressed as means ± SEM. *P<0.05; **P<0.01; ***P<0.001; ****P<0.0001. N.S., statistically not significant, with the comparisons labeled.

**Figure 5:**
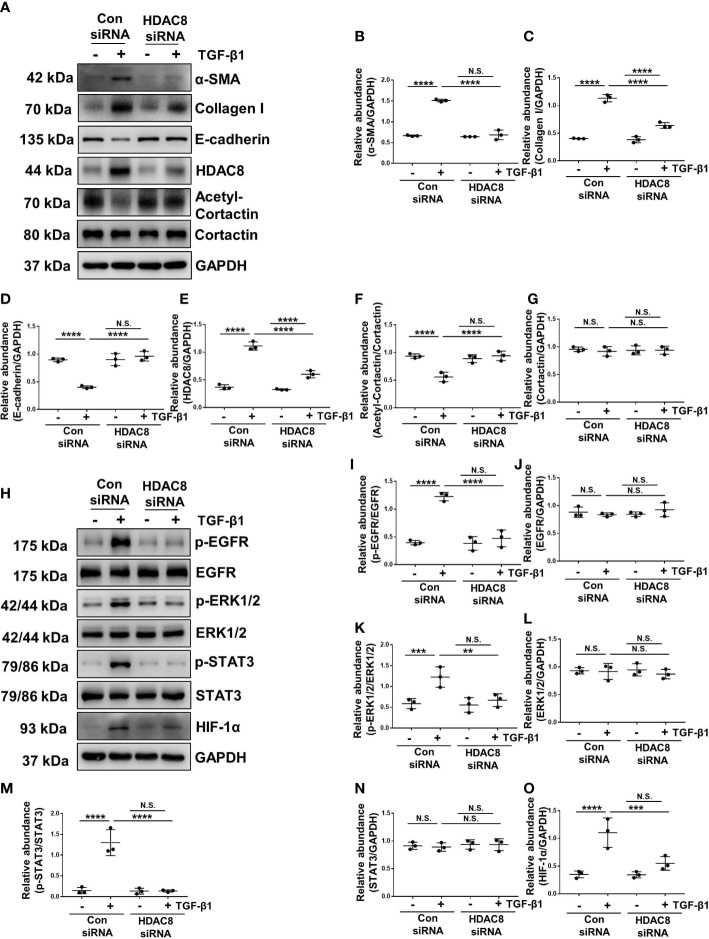
Blockade of HDAC8 with siRNA abrogates EMT by inhibition of EGFR/ERK1/2/STAT3/HIF-1α signaling pathway **(A)** Serum-starved HPMCs were pretreated with siRNA and then exposed to TGF-β1 (2 ng/ml) for 36 h. Cell lysates were subjected to immunoblot analysis with specific antibodies against α-SMA, collagen I, E-cadherin, HDAC8, cortactin, acetyl-cortactin and GAPDH. Expression levels of **(B)** α-SMA, **(C)** collagen I, **(D)** E-cadherin, **(E)** HDAC8, **(F)** acetyl-cortactin, **(G)** cortactin in different groups were quantified by densitometry and normalized with GAPDH and cortactin respectively. **(H)** Cell lysates were subjected to immunoblot analysis with specific antibodies against p-EGFR, EGFR, p-ERK1/2, ERK1/2, p-STAT3, STAT3, HIF-1α and GAPDH. Expression levels of **(I)** p-EGFR, **(J)** EGFR, **(K)** p-ERK1/2, **(L)** ERK1/2, **(M)** p-STAT3, **(N)** STAT3, **(O)** HIF-1α were quantified by densitometry and normalized with GAPDH, EGFR, ERK1/2 and STAT3 respectively. Data were expressed as means ± SEM. *P<0.05; **P<0.01; ***P<0.001; ****P<0.0001. N.S., statistically not significant, with the comparisons labeled.

TGF-β1 is an important cytokine that can stimulate EMT and induce peritoneal fibrosis (7). HPMCs exposure to TGF-β1 increased the expression of α-SMA and collagen I, and decreased the expression of epithelial cell marker E-cadherin, indicating that TGF-β1 promoted the EMT of HPMCs (**Figures 4A–D**, **5A–D**). Treatment of PCI-34051 or siRNA, decreased the expression of HDAC8 and increased the expression of acetyl-cortactin (**Figures 4A, E–G**, **5A, E–G**). In addition, TGF-β1 induced the phosphorylation of EGFR and activation of its downstream signaling pathways ERK1/2 and STAT3/HIF-1α (**Figures 4H–O**, **5H–O**). Blockade of HDAC8 suppressed all of these responses. These data supported our *in vivo* observation that HDAC8 is a key protein in regulating peritoneal fibrosis *via* EGFR/ERK1/2/STAT3/HIF-1α signaling pathway.

### Inhibition of HDAC8 prevents M2 macrophage polarization *via* suppressing STAT6 and PI3K/Akt signaling pathways in the high glucose PDF-injured peritoneum

**Figure 6:**
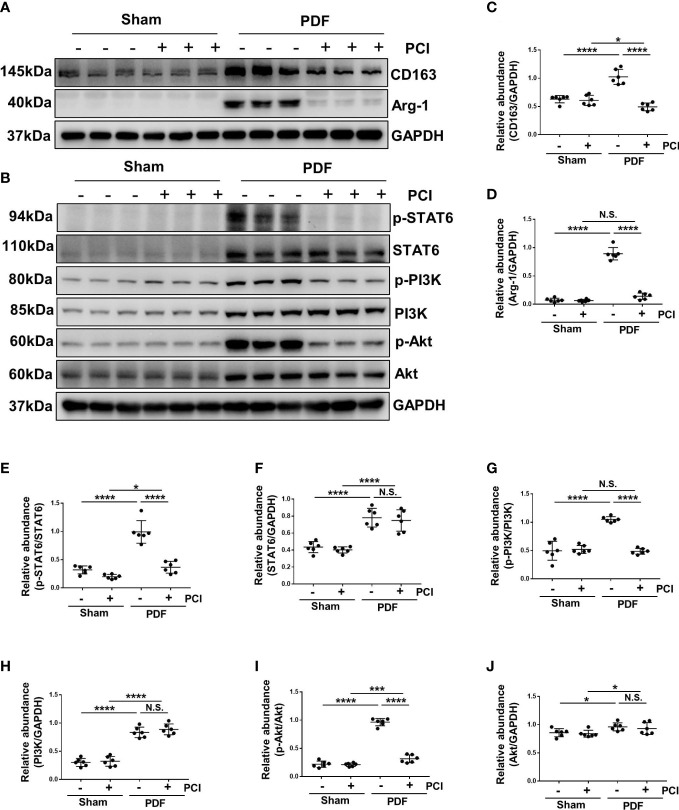
Inhibition of HDAC8 prevents M2 macrophage polarization *via* suppressing STAT6 and PI3K/Akt signaling pathways in the high glucose PDF-injured peritoneum **(A)** Western blot analysis showed the protein levels of CD163, Arg-1 and GAPDH in peritoneum of mice. **(B)** Western blot analysis showed the protein levels of p-STAT6, STAT6, p-PI3K, PI3K p-Akt, Akt and GAPDH in peritoneum of mice. Expression levels of **(C)** CD163, **(D)** Arg-1, **(E)** p-STAT6, **(F)** STAT6, **(G)** p-PI3K, **(H)** PI3K, **(I)** p-Akt, **(J)** Akt were quantified by densitometry and normalized with GAPDH, STAT6, PI3K and Akt respectively. Data were expressed as means ± SEM. *P<0.05; **P<0.01; ***P<0.001; ****P<0.0001. N.S., statistically not significant, with the comparisons labeled.

Macrophages play a pivotal role in peritoneal fibrosis process (13). Therefore, we detected the infiltration and polarization of macrophages in a mouse peritoneal fibrosis model established by 4.25% glucose PDF. Western blot showed increased expressions of arginase-1 (Arg-1) and CD163 expression [cell markers of M2 phenotype (13)] after exposure to 4.25% glucose PDF, while PCI-34051 administration effectively decreased their expressions (**Figures 6A, C, D**). These results suggested that M2 macrophage polarization was involved in the process of peritoneal fibrosis, and the inhibition of HDAC8 by PCI-34051 could prevent M2 macrophage polarization.

The phosphorylation of STAT6 and activation of PI3K/AKT signaling pathway are involved in the regulation of M2 macrophage polarization (13). In our research, the phosphorylation of STAT6, PI3K and Akt were significantly increased after exposure to 4.25% glucose PDF, while PCI-34051 administration inhibited their phosphorylation (**Figures 6B, E, G, I**). In addition, injection of 4.25% glucose PDF increased the expression of total STAT6, PI3K and Akt, and PCI-34051 administration did not decrease their expressions (**Figures 6B, E–J**). These data suggested that inhibition of HDAC8 might inhibit the M2 macrophage polarization *via* STAT6 and PI3K/Akt pathways.

### Blockade of HDAC8 with PCI-34051 or siRNA prevents M2 macrophage polarization *via* suppressing STAT6 and PI3K/Akt signaling pathways

**Figure 7:**
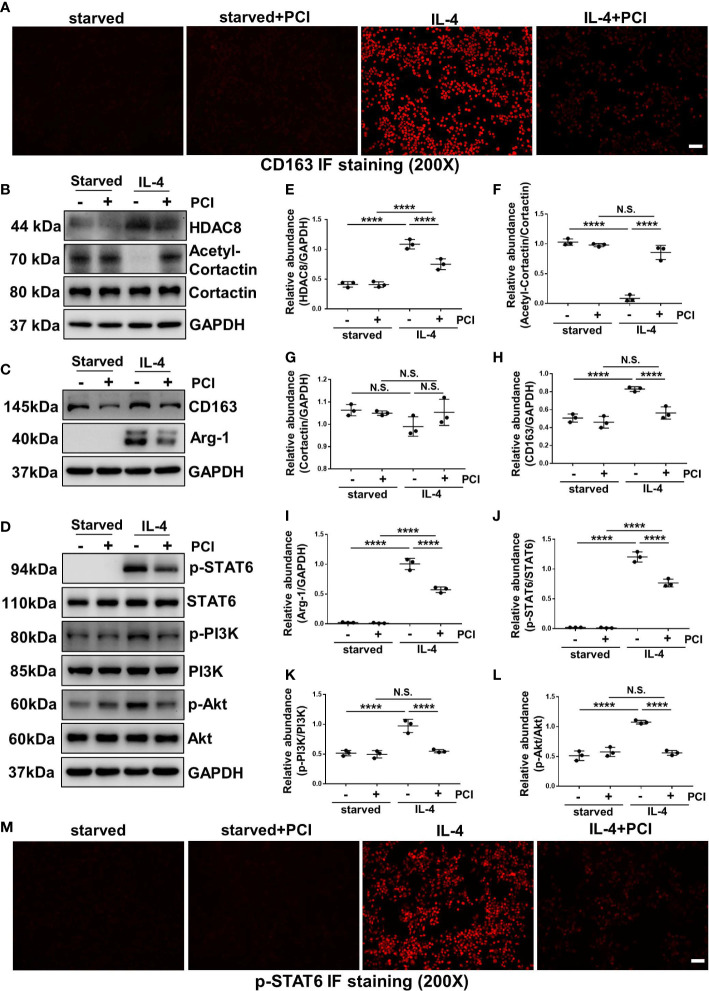
Blockade of HDAC8 with PCI-34051 prevents M2 macrophage polarization *via* suppressing STAT6 and PI3K/Akt signaling pathways **(A)** Immunofluorescent staining of CD163 in Raw264.7 cells with different treatments **(B–D)** Serum-starved RAW264.7 were pretreated with PCI-34051 (5μM) and then exposed to IL-4 (10 ng/ml) for 24 h. Cell lysates were subjected to immunoblot analysis with specific antibodies against HDAC8, acetyl-cortactin, cortactin, CD163, Arg-1, p-STAT6, STAT6, p-PI3K, PI3K p-Akt, Akt and GAPDH. Expression levels of **(E)** HDAC8, **(F)** acetyl-cortactin, **(G)** cortactin, **(H)** CD163, **(I)** Arg-1, **(J)** p-STAT6, **(K)** p-PI3K, **(L)** p-Akt were quantified by densitometry and normalized with GAPDH, cortactin, STAT6, PI3K and Akt respectively. **(M)** Immunofluorescent staining of p-STAT6 in Raw264.7 cells with different treatments. Data were expressed as means ± SEM. *P<0.05; **P<0.01; ***P<0.001; ****P<0.0001. N.S., statistically not significant, with the comparisons labeled. All scale bars = 50 μm.

**Figure 8:**
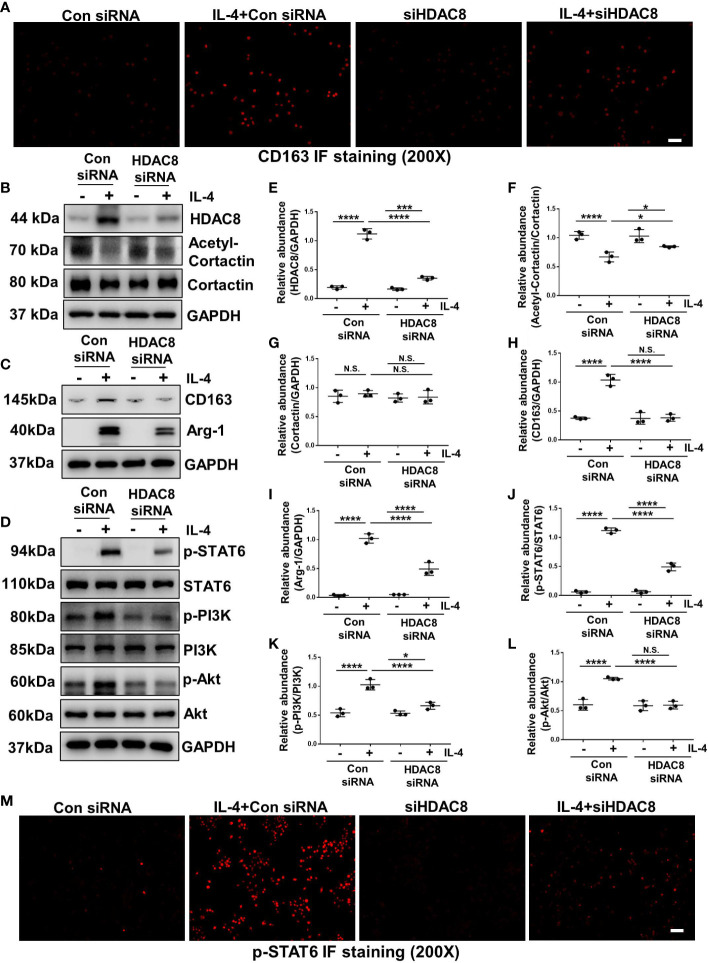
Blockade of HDAC8 with siRNA prevents M2 macrophage polarization *via* suppressing STAT6 and PI3K/Akt signaling pathways **(A)** Immunofluorescent staining of CD163 in Raw264.7 cells with different treatments **(B–D)** Serum-starved RAW264.7 were pretreated with siRNA and then exposed to IL-4 (10 ng/ml) for 24 h. Cell lysates were subjected to immunoblot analysis with specific antibodies against HDAC8, acetyl-cortactin, cortactin, CD163, Arg-1, p-STAT6, STAT6, p-PI3K, PI3K p-Akt, Akt and GAPDH. Expression levels of **(E)** HDAC8, **(F)** acetyl-cortactin, **(G)** cortactin, **(H)** CD163, **(I)** Arg-1, **(J)** p-STAT6, **(K)** p-PI3K, **(L)** p-Akt were quantified by densitometry and normalized with GAPDH, cortactin, STAT6, PI3K and Akt respectively. **(M)** Immunofluorescent staining of p-STAT6 in Raw264.7 cells with different treatments. Data were expressed as means ± SEM. *P<0.05; **P<0.01; ***P<0.001; ****P<0.0001. N.S., statistically not significant, with the comparisons labeled. All scale bars = 50 μm.

IL-4 stimulation can induce M2 macrophage polarization (13). As shown in **Figures 7A** and **8A**, immunofluorescent staining of CD163 indicated that IL-4 induced M2 polarization in RAW264.7 cells, while blockade of HDAC8 prevented M2 macrophage polarization. Western blot further verified this result (**Figures 7B, C, E–I**, **8B, C, E–I**). In addition, IL-4 induced the phosphorylation of STAT6 and activation of PI3K/Akt pathway (**Figures 7D, J–L**, **8D, J–L**, **S2**, **S3**). Blockade of HDAC8 with PCI-34051 or siRNA suppressed all of these responses. Immunofluorescent staining of p-STAT6 further verified that blockade of HDAC8 could suppress the phosphorylation of STAT6 (**Figures 7M**, **8M**). These data supported our *in vivo* observation that HDAC8 could regulate M2 macrophage polarization *via* STAT6 and PI3K/Akt signaling pathways.

### Inhibition of HDAC8 reduces cell apoptosis *in vivo* and vitro

BAX and cleaved-caspase-3 are both pivotal proteins involved in cell apoptosis (24, 25). *In vivo* and *in vitro* experiments (**Figure S4**), we demonstrated that exposure to 4.25% glucose PDF or TGF-β1 stimulation would promote the cell apoptosis, while blockade of HDAC8 could reduce cell apoptosis.

## Discussion

Peritoneal fibrosis is one of the most significant complications for PD patients, however, up to now there is no effective solution (3, 26). In this research, we demonstrated that inhibition of HDAC8 with PCI-34051 or siRNA suppressed EMT, apoptosis and M2 macrophage polarization, ultimately attenuated peritoneal fibrosis and peritoneal dysfunction. Thus, inhibition of HDAC8 may be a potential therapeutic strategy for prevention and treatment of peritoneal fibrosis in long term PD patients.

HDAC8 is a sex-linked gene located at chromosomal position Xq13.1 (27) and expressed both in nucleus and cytoplasm (28–30). HDAC8 has a variety of histone and non-histone (SMC3, α-tubulin, cortactin, HSP20, p53, PKM2, AKT, ERRα and c-Jun) substrates (31–34). As one of the target substrates of HDAC8, cortactin is a ubiquitous multidomain protein involved in the regulation of actin cytoskeleton, integrin signaling and ECM degradation (35). Acetyl-cortactin is localized in the nucleus and can be stimulated by growth factors to transport into the cytoplasm (36, 37). In our study, we found that HDAC8 expression was increased in peritoneum stimulated by high glucose PDF or HPMCs exposed to TGF-β1, and overexpression of HDAC8 inhibited the acetylation of cortactin. Since cortactin is only expressed in the cytoplasm of cells (37), we hypothesized that HDAC8 regulated peritoneal fibrosis by participating in cytoplasmic protein regulation.

EGFR is a tyrosine kinase receptor that binds to ligands and phosphorylates, subsequently leading to the activation of several signaling pathways, including ERK1/2 and STAT3 (8). Our previous studies have demonstrated that activated EGFR promotes peritoneal fibrosis by regulating EMT, inflammation, and angiogenesis (8). In addition, previous studies have demonstrated that EGFR activation is regulated by several histone deacetylases, including HDAC1 (38), HDAC4 (39) and HDAC6 (40, 41). In this research, we found that overexpression of HDAC8 could promote the phosphorylation of EGFR and the activation of its downstream signal molecules. Although the mechanism by which HDAC8 regulates EGFR is still unknown, we speculate that HDAC8 may act by affecting the endocytic trafficking and degradation of EGFR. Activation of EGFR signaling is terminated by endocytosis, and vesicles containing the receptor-ligand complex target lysosomes for degradation along microtubule tracks (42). Acetylation of microtubule component α-tubulin affects the stability of microtubule and further regulates intracellular cargo (such as EGFR-containing vesicles) transport (42). The deacetylation of α-tubulin is mainly mediated by HDAC6, but HDAC8 has also recently been characterized to be involved in the deacetylation of α-tubulin (43). HDAC8 is significantly overexpressed in HeLa cells and may take over the function of HDAC6 as a major deacetylase of α-tubulin (43). Therefore, we speculate that HDAC8 might inhibit EGFR endocytosis by deacetylating α-tubulin in the cytoplasm, and result in the continuous activation of EGFR.

M2 macrophages are believed to be involved in peritoneal fibrosis, and consumption of M2 macrophages in the peritoneum can alleviate peritoneal fibrosis (44). Our study found that blockade of HDAC8 could prevent M2 macrophage polarization *via* STAT6 and PI3K/Akt signaling pathways. STAT6 is a major factor in the M2 polarization process of macrophages, and its activation drives M2 polarization (45). Recent studies have shown that acetylation of STAT6 can inhibit its transcriptional activity and thus inhibit M2 polarization (46). Although the specific mechanism by which HDAC8 regulates STAT6 has not been reported, several studies have demonstrated the direct regulatory effect of HDACs on STAT transcription factors. A recent study has demonstrated that IL-4-STAT6 signaling is dependent on HDAC3, which performs post-translational modifications and allosteric regulation of STAT6 by occupying STAT6-repressed enhancers (47). The expression and phosphorylation of STAT3 are also regulated by several HDACs, including HDAC1 and HDAC3 (48, 49). Whether HDAC8 affects the activation of STAT6 through acetylation remains to be further investigated. The PI3K/Akt pathway can influence the survival, migration and polarization of macrophages (50). It has been demonstrated that HDAC8 induced tri-methylation of histone H3 lysine 27 through down-regulating the H3K27me3 eraser Jumonji Domain Containing 3 could suppress PTEN expression, thus activating the PI3K/Akt signaling pathway, and further determining susceptibility to cell cycle arrest induced by anthrax lethal toxin (51). In this research, we also found that knockdown of HDAC8 could suppress the activation of PI3K/Akt signaling pathway. The regulatory effect of HDA8 on Akt, on one hand, is mediated by its upstream signaling molecule PI3K; On the other hand, Soon-Duck Ha et al. have demonstrated that HDAC8 activates Akt through upregulating PLCB1 and suppressing DESC1 expression (52). In conclusion, targeting HDAC8 could further regulate M2 macrophage polarization.

At present, the development of HDAC8 inhibitors focuses on improving their activity and high selectivity. Meanwhile, the multi-target pharmacological approach on HDAC8 has gained attention for its benefits from achieving the simultaneous modulation of multiple targets, especially in complex diseases such as cancer and fibrosis (53, 54). PCI-34051 is currently the most widely used HDAC8 inhibitor in the validation of various target diseases due to its considerable subtype selectivity. PCI-34051 in combination with conventional antitumor agents has shown to have a synergistic effect in the reversal of disease (55, 56). Whether the combination of PCI-34051 and other drugs could play a reverse role in fibrosis diseases, which remains to be further studied.

In conclusion, we demonstrated that blockade of HDAC8 could prevent and reverse peritoneal fibrosis. Mechanistically, HDAC8 promoted EMT by inducing the phosphorylation of EGFR and in turn led to the activation of its downstream fibrotic signaling pathways, including STAT3/HIF-1α and ERK1/2. HDAC8 is also a key protein in regulating M2 macrophage polarization *via* STAT6 and PI3K/Akt signaling pathways (**Figure S5**). As such, targeting HDAC8 might be a new strategy to lessen the severity of peritoneal fibrosis.

## Data availability statement

The original contributions presented in the study are included in the article/**Supplementary Materials**. Further inquiries can be directed to the corresponding author.

## Ethics statement

This study was approved by the Medical Ethics Committee of Shanghai East Hospital and was conducted in accordance with the Declaration of Helsinki. Written informed consent was obtained from each patient. And we have obtained the registration number from the Chinese Clinical Trial Register (ChiCTR): ChiCTR2100052103. The animal protocol was reviewed and approved by the Institutional Animal Care and Use Committee at Tongji University (Shanghai, China).

## Author contributions

NL participated in research design. XZ, HC, YS, JL, XM, LD, YH, MT, QZ and DY conducted experiments. XZ, HC, YS and NL contributed new reagents or analytic tools. XZ performed data analysis. XZ, SZ and NL wrote or contributed to the writing of the manuscript. All authors read and approved the final version of the article.
