# Supplementary material for: Histone deacetylase 8 inhibition prevents the progression of peritoneal fibrosis by counteracting the epithelial-mesenchymal transition and blockade of M2 macrophage polarization
Source: Front Immunol. 2023 Feb 23;14:1137332. doi: 10.3389/fimmu.2023.1137332 (PMC9995794; doi:10.3389/fimmu.2023.1137332)
Supplement: Supplementary file 1 [file DataSheet_1.pdf]

# Supplementary Materials and Methods

## Histone deacetylase 8 inhibition prevents the progression of peritoneal fibrosis by counteracting the epithelial-mesenchymal transition and blockade of M2 macrophage polarization

Xun Zhou[1], Hui Chen[1], Yingfeng Shi[1], Jinqing Li[1], Xiaoyan Ma[1], Lin Du[1], Yan Hu[1], Min Tao [1], Qin Zhong[1], Danying Yan[1], Shougang Zhuang[1,2], Na Liu[1]

[1]Department of Nephrology, Shanghai East Hospital, Tongji University School of Medicine, Shanghai, China;

[2]Department of Medicine, Rhode Island Hospital and Alpert Medical School, Brown University, Providence, RI, USA

**Correspondence and offprint requests to: Na Liu**, M.D., Ph.D., Department of Nephrology, Shanghai East Hospital, Tongji University School of Medicine, 150 Jimo road, Pudong new district, Shanghai 200120, China. **E-mail**: naliubrown@163.com.

**Reagents and kits**

PCI-34051 was purchased from Selleckchem (Houston, TX, United States). FITC and Texas Red for immunofluorescent staining were purchased from Thermo Fisher Scientific (Waltham, MA, United States). Enzyme-linked immunosorbent assay (ELISA) kits for HDAC8 was purchased from Shanghai Enzyme-linked Biotechnology (Shanghai, China); TGF-β1, VEGF and CA125 were purchased from Wuhan Cloud-Clone Corp. (Wuhan, China). BUN and glucose biochemical reagent kits were purchased from Nanjing Jiancheng Bioengineering Institute (Nanjing, China). Peritoneal dialysis fluid with 4.25% glucose was purchased from Baxter Healthcare (Guangzhou, China). HDAC8 siRNA were purchased from GenePharma (Shanghai, China). Antibodies to p-EGFR (Tyr1068) (#3777), EGFR (#4267), ERK1/2 (#4695), p-ERK1/2 (#4370), PI3K (#4257), p-PI3K (#17366), AKT (#4691), p-AKT (#4060), STAT3 (#9139), p-STAT3 (#9138), E-cadherin (#14472), STAT6 (#5397) and p-STAT6 (#56554) were purchased from Cell Signaling Technology (Danvers, MA, United States). Antibodies to HIF-1α (ab228649) was purchased from Abcam (Cambridge, MA, United States). Antibodies to GAPDH (sc-32233), HDAC8 (sc-17778), cortactin (sc-55579) and Collagen I (A2) (sc-28654) were purchased from Santa Cruz Biotechnology (San Diego, CA, United States). Antibodies to Arginase-1 (GB11285) and CD163 (GB11340) were purchased from Servicebio (Wuhan, China). Antibodies to acetyl-cortactin (lys309) (ABT1378) was purchased from EMD Millipore (Temecula, CA, United States). IL-4 and TGF-β1were purchased from R&D Systems (Minneapolis, MN, United States). Antibody to α-SMA (A2547) and all other chemicals were obtained from Sigma-Aldrich (St. Louis, MO, United States).

**Animals and treatment**

Male C57BL mice (purchased from Shanghai Super-B&K Laboratory Animal Corp. Ltd) weighed 20-25g were used in this study. The peritoneal fibrosis model was created by daily i.p. injection of 100 ml/kg peritoneal dialysis fluid with 4.25% glucose for 28 days [1]. To investigate the effect of PCI-34051 in peritoneal fibrosis, mice were injected i.p. with a single dose of PCI-34051 (20mg/kg) in DMSO every day. Mice were

randomly allocated into four groups for each model: (1) mice injected with an equivalent amount of saline i.p. and DMSO (n=6), defined as the Sham group, (2) mice injected an equivalent amount of saline i.p. and PCI-34051 (n=6), defined as Sham + PCI-34051 group, (3) mice injected with PDF and DMSO (n=6), defined as PDF group, and (4) mice injected PDF and PCI-34051, defined as PDF + PCI-34051 group (n=6). At the end of 28 days, all mice were killed by exsanguination under anesthesia with inhaled 5% isoflurane in room air and the parietal peritoneum was collected from each mouse for further experiments. The animal protocol was reviewed and approved by the Institutional Animal Care and Use Committee at Tongji University (Shanghai, China).

**Transfection of siRNA**

Transfection of siRNA was performed according to the manufacturer's protocol, respectively. Cells were seeded to 30-40% confluence in the antibiotic-free medium and grown followed by transfection with siRNA (60 pmol) using Lipofectamine 3000. In parallel, scrambled siRNA (60 pmol) was used as a control for off-target changes in cells. 6 hours after transfection, the medium was changed to DMEM with F12 or RPMI-1640 containing 0.5% FBS for starvation and then cells were incubated with or without TGF-β1 (2ng/ml) or IL-4 (10 ng/ml) for an additional 36 hours or 24 hours before being harvested for analysis. All of the in vitro experiments were repeated for at least three times.

**Peritoneal equilibration test**

Mice were injected with 2 ml 4.25% PDF for two hours and then euthanized to collect blood and dialysate. Peritoneal fluid (D0) was collected immediately from the mice, while the peritoneal fluid (D2) and plasma (P2) were collected 2 hours after injection of PDF. According to the manufacturer's instructions, we used biochemical reagent kits to determine glucose in dialysate and BUN in plasma and dialysate. Functional alteration of the peritoneal membrane was evaluated by the urea nitrogen transport rate from plasma with the dialysate-to-plasma (D/P) ratio of blood urea nitrogen, and the glucose absorption rate from dialysate with the ratio of dialysate glucose at 2 hours after

PDF injection to dialysate glucose at 0 hours (D/D0).

**Enzyme-linked immunosorbent assay (ELISA) analysis**

ELISA detection of HDAC8, TGF-β1, VEGF and CA125 proteins in human PD effluents were performed following the manufacturer's instructions.

**Morphologic studies of peritoneum**

All specimens were fixed in 4% formaldehyde and embedded in paraffin blocks. Sections (3-μm-thick) were performed with Masson trichrome staining to evaluate peritoneal fibrosis. Masson trichrome staining was performed according to the protocol provided by the manufacturers. The thickness of the sub-mesothelial tissue was evaluated (in micrometers), and the average of ten independent measurements was calculated for each section (original magnification: 200X).

**Immunofluorescent staining**

Immunofluorescent staining was carried out according to the procedure described in our previous study [2]. For immunofluorescent staining, FPE sections (3 μm) were rehydrated and incubated with primary antibodies against α-SMA (1:100), EGFR (1:200), CD163 (1:500), p-STAT6 (1:400) or HDAC8 (1:200) and then Texas Red- or FITC-labeled secondary antibodies. The densitometry analysis of immunoblot results was conducted by using ImageJ software (National Institutes of Health, Bethesda, MD, United States). For cell immunostaining, cells cultured on coverslips were washed with PBS, fixed with 4% paraformaldehyde, permeabilized with 0.1% (vol/vol) Triton X-100 and 0.1 mM glycine, and then incubated 30 min in PBS containing 5% serum. Cells were then treated with primary antibodies at room temperature for 1h. After washing with PBS, cells were incubated with a mixture of FITC-labeled goat anti-rabbit IgG antibody for 1h at room temperature.

**Immunoblotting analysis**

Immunoblotting analysis was carried out according to the procedure described in our

previous study [2]. Cell samples and peritoneal tissue samples were prepared and determined total protein concentration (μg/μl) for clarified homogenates by bicinchoninic acid (BCA) assay, according to the manufacturer's instructions (ThermoFisher). Proteins were separated by SDS-PAGE Gel electrophoresis (8%-12%) in 120V for 90 minutes and transferred to 0.2 mm nitrocellulose membranes (80V for 80 minutes). After incubation with 5% nonfat milk for 1 h at room temperature, the membranes were incubated with primary antibodies overnight at 4°C and then incubated with appropriate horseradish peroxidase-conjugated secondary antibodies for 1 h on the shaker at room temperature. Membranes were washed three times with prewarmed TBST in 10 minutes. Bound antibodies were visualized by chemiluminescence detection. Densitometry analysis of immunoblot results was conducted by using Image J software (National Institutes of Health, Bethesda, MD, USA).

**Statistical analysis.**

All of the experiments were conducted at least three times. Data depicted in graphs represented the means ± SEM for each group. One-way analysis of variance (ANOVA) followed by Tukey's post-test was used for multiple comparisons. Pearson correlation analysis was used to study the bivariate normal distribution, and Spearman correlation analysis for non-normal distribution data. All tests were two-tailed, and $P<0.05$ was considered statistically significant. The statistical analyses were conducted by using IBM SPSS Statistics 20.0 (Beijing, China).

**References:**

1. Xu L, Liu N, Gu H, Wang H, Shi Y, Ma X *et al*. Histone deacetylase 6 inhibition counteracts the epithelial-mesenchymal transition of peritoneal mesothelial cells and prevents peritoneal fibrosis. Oncotarget (2017) 8: 88730-88750.

2. Pang M, Kothapally J, Mao H, Tolbert E, Ponnusamy M, Chin YE *et al*. Inhibition of histone deacetylase activity attenuates renal fibroblast activation and interstitial fibrosis in obstructive nephropathy. Am J Physiol Renal Physiol (2009) 297: F996-

F1005.
