# Supplementary material for: Histone deacetylase 8 inhibition prevents the progression of peritoneal fibrosis by counteracting the epithelial-mesenchymal transition and blockade of M2 macrophage polarization
Source: Front Immunol. 2023 Feb 23;14:1137332. doi: 10.3389/fimmu.2023.1137332 (PMC9995794; doi:10.3389/fimmu.2023.1137332)
Supplement: Supplementary file 2 [file DataSheet_2.pdf]

# Supplementary Figures

**Histone deacetylase 8 inhibition prevents the progression of peritoneal fibrosis by counteracting the epithelial-mesenchymal transition and blockade of M2 macrophage polarization**

Xun Zhou[1], Hui Chen[1], Yingfeng Shi[1], Jinqing Li[1], Xiaoyan Ma[1], Lin Du[1], Yan Hu[1], Min Tao [1], Qin Zhong[1], Danying Yan[1], Shougang Zhuang[1,2], Na Liu[1]

[1]Department of Nephrology, Shanghai East Hospital, Tongji University School of Medicine, Shanghai, China;

[2]Department of Medicine, Rhode Island Hospital and Alpert Medical School, Brown University, Providence, RI, USA

**Correspondence and offprint requests to: Na Liu**, M.D., Ph.D., Department of Nephrology, Shanghai East Hospital, Tongji University School of Medicine, 150 Jimo road, Pudong new district, Shanghai 200120, China. **E-mail**: naliubrown@163.com.

Figure S1

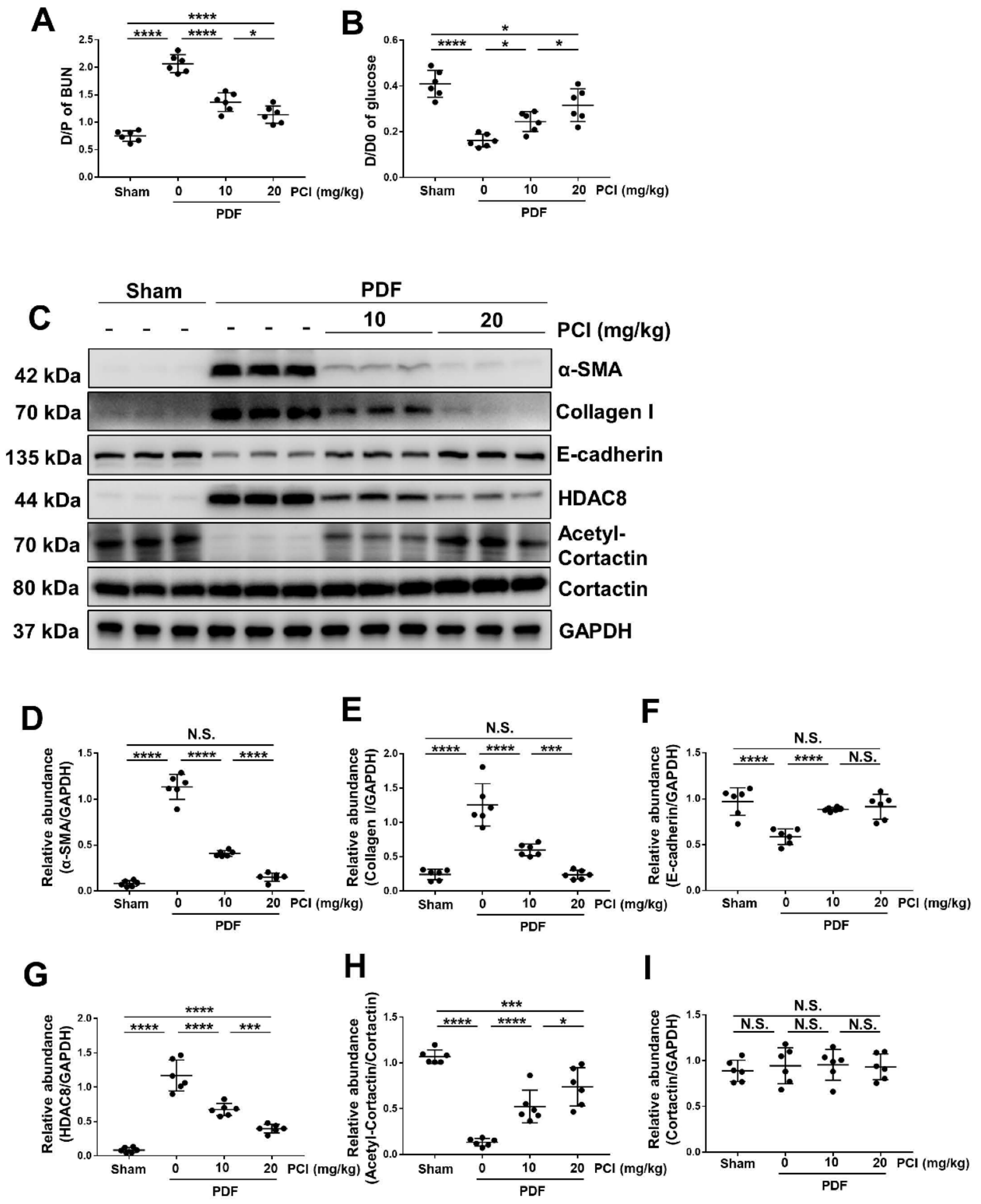


**Figure S1 Effect of different concentrations of PCI-34051 on peritoneal fibrosis induced by high glucose PDF**

(A) The dialysate-to-plasma (D/P) ratio of blood urea nitrogen (BUN). (B) Ratio of dialysate glucose at 2 h after PDF injection to dialysate glucose at 0 hour (D/D0). (C)

Western blot analysis showed the protein levels of α-SMA, collagen I, E-cadherin, HDAC8, cortactin, acetyl-cortactin and GAPDH in peritoneum from different groups of mice. Expression levels of (D) α-SMA, (E) collagen I, (F) E-cadherin, (G) HDAC8, (H) acetyl-cortactin, (I) cortactin in different groups were quantified by densitometry and normalized with GAPDH and cortactin respectively. Data were expressed as means ± SEM. *$P<0.05$; **$P<0.01$; ***$P<0.001$; ****$P<0.0001$. N.S., statistically not significant, with the comparisons labeled.

## Figure S2

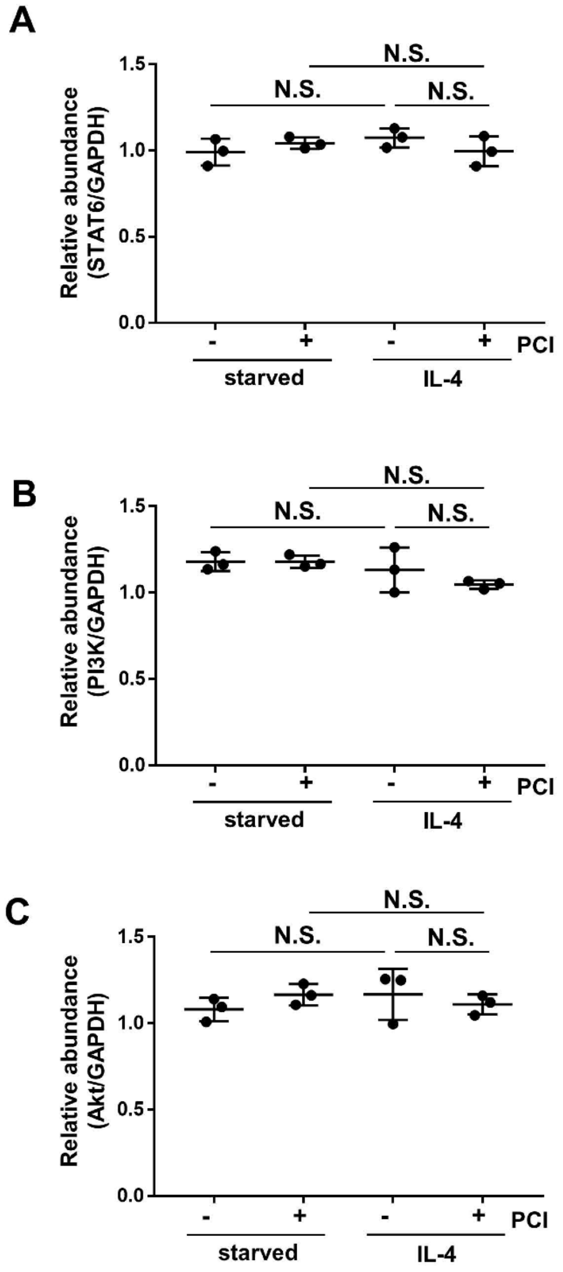


**Figure S2 Quantitative analysis of STAT6, PI3K and Akt in macrophage stimulated with IL-4 and treated with PCI-34051**

Expression levels of (A) STAT6, (B) PI3K, (C) Akt in different groups were quantified by densitometry and normalized with GAPDH respectively. Data were expressed as

means ± SEM. *$P$<0.05; **$P$<0.01; ***$P$<0.001; ****$P$<0.0001. N.S., statistically not significant, with the comparisons labeled.

**Figure S3**

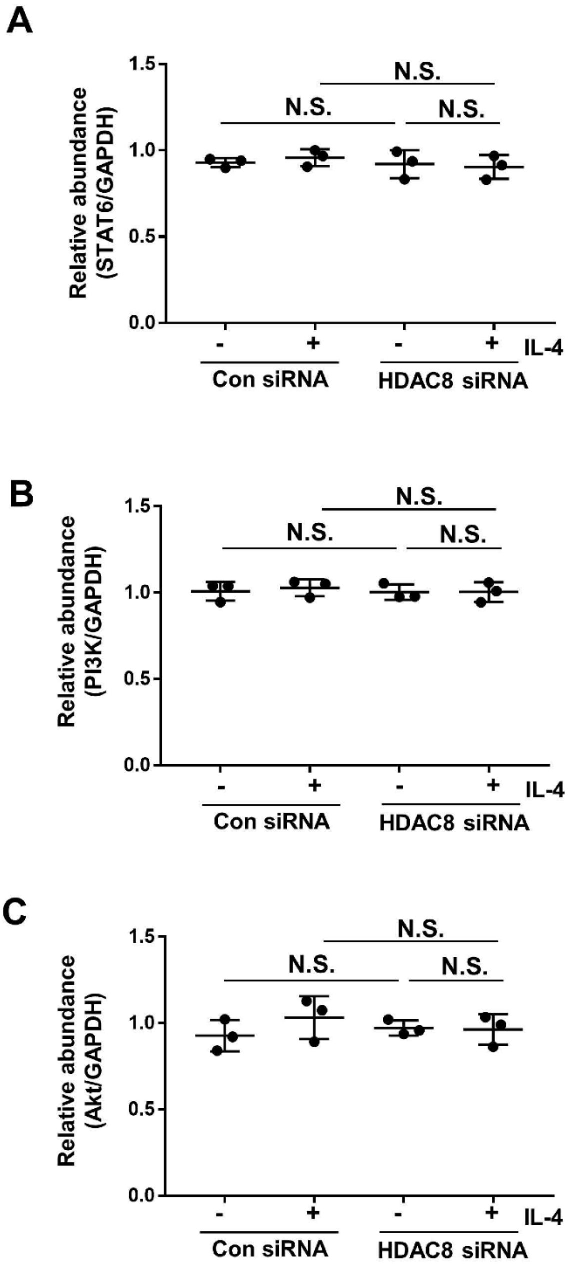


**Figure S3 Quantitative analysis of STAT6, PI3K and Akt in macrophage stimulated with IL-4 and treated with siRNA**

Expression levels of (A) STAT6, (B) PI3K, (C) Akt in different groups were quantified by densitometry and normalized with GAPDH respectively. Data were expressed as

means ± SEM. *$P<0.05$; **$P<0.01$; ***$P<0.001$; ****$P<0.0001$. N.S., statistically not significant, with the comparisons labeled.

# Figure S4

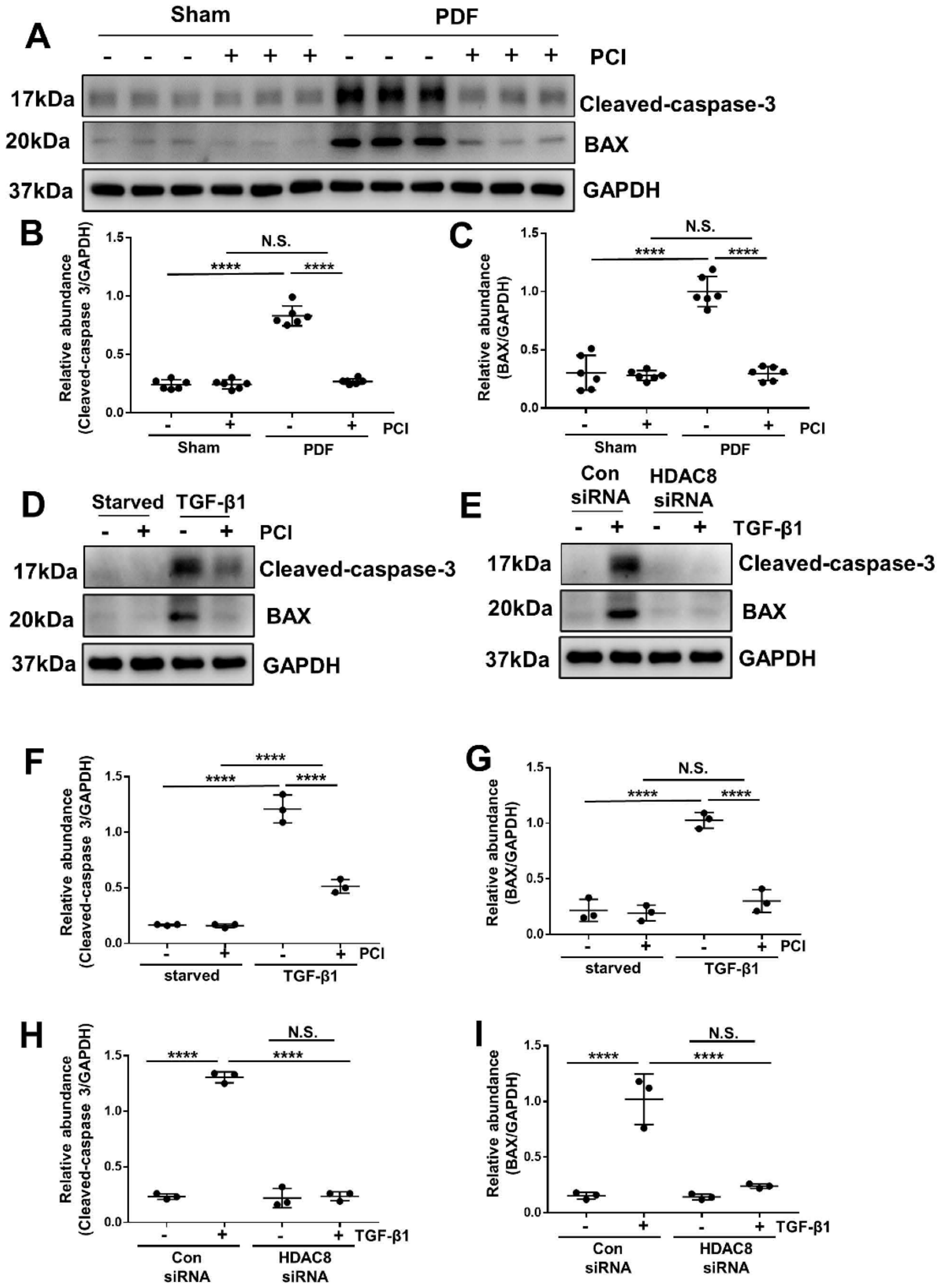


**Figure S4 Inhibition of HDAC8 reduces cell apoptosis in vivo and vitro**

(A) Western blot analysis showed the protein levels of cleaved-caspase-3, BAX and GAPDH in peritoneum from different groups of mice. Expression levels of (B) cleaved-caspase-3, (C) BAX in different groups were quantified by densitometry and

normalized with GAPDH respectively. (D-E) Serum-starved HPMCs were pretreated with PCI-34051 (5μM) or siRNA and then exposed to TGF-β1 (2 ng/ml) for 36 h. Cell lysates were subjected to immunoblot analysis with specific antibodies against cleaved-caspase-3, BAX and GAPDH. Expression levels of (F, H) cleaved-caspase-3, (G, I) BAX in different groups were quantified by densitometry and normalized with GAPDH respectively. Data were expressed as means ± SEM. *$P$<0.05; **$P$<0.01; ***$P$<0.001; ****$P$<0.0001. N.S., statistically not significant, with the comparisons labeled. All scale bars = 50 μm.

Figure S5

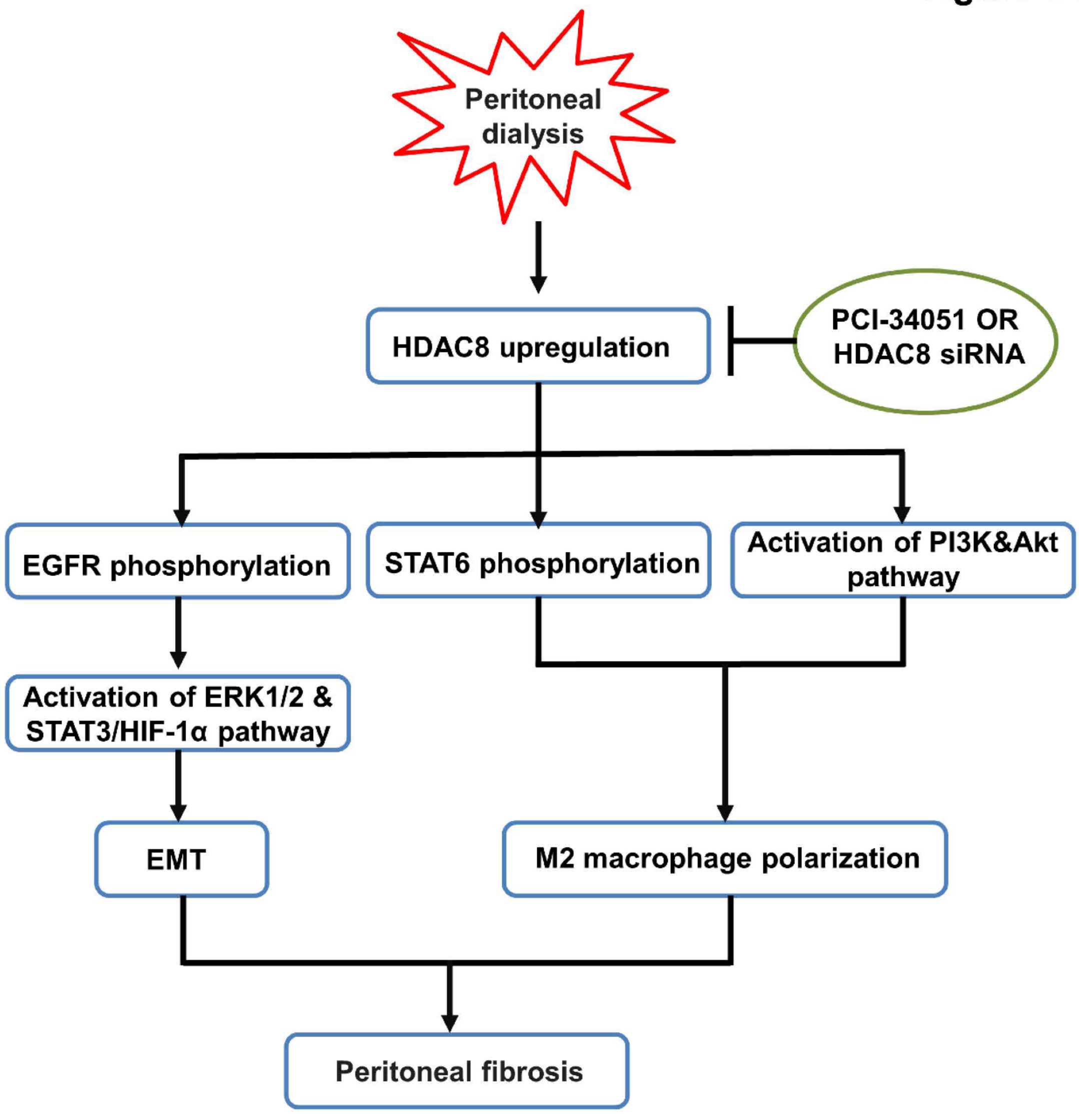


**Figure S5 Mechanism of HDAC8 in peritoneal fibrosis progression**

HDAC8 was upregulated in peritoneum exposed to high glucose dialysate, which subsequently leaded to EMT through activation of profibrotic signaling pathway (EGFR/ERK1/2/STAT3/HIF-1α) and induction of M2 macrophage polarization via

STAT6 and PI3K/Akt pathways. All these responses could be inhibited by PCI-34051 or HDAC8 siRNA. Abbreviations: HDAC8, histone deacetylases 8; EGFR, epidermal growth factor receptor; ERK 1/2, extracellular signal-regulated kinases 1/2; STAT3, signal transducer and activator of transcription 3; PI3K, phosphatidylinositol-3-kinase.
