# Supplementary material for: Histone deacetylase 8 inhibition prevents the progression of peritoneal fibrosis by counteracting the epithelial-mesenchymal transition and blockade of M2 macrophage polarization
Source: Front Immunol. 2023 Feb 23;14:1137332. doi: 10.3389/fimmu.2023.1137332 (PMC9995794; doi:10.3389/fimmu.2023.1137332)
Supplement: Supplementary file 3 [file Table_1.pdf]

Supplementary Table

Histone deacetylase 8 inhibition prevents the progression of peritoneal fibrosis by counteracting the epithelial-mesenchymal transition and blockade of M2 macrophage polarization

Xun Zhou[1], Hui Chen[1], Yingfeng Shi[1], Jinqing Li[1], Xiaoyan Ma[1], Lin Du[1], Yan Hu[1], Min Tao [1], Qin Zhong[1], Danying Yan[1], Shougang Zhuang[1,2], Na Liu[1]

[1]Department of Nephrology, Shanghai East Hospital, Tongji University School of Medicine, Shanghai, China;

[2]Department of Medicine, Rhode Island Hospital and Alpert Medical School, Brown University, Providence, RI, USA

Correspondence and offprint requests to: Na Liu, M.D., Ph.D., Department of Nephrology, Shanghai East Hospital, Tongji University School of Medicine, 150 Jimo road, Pudong new district, Shanghai 200120, China. **E-mail**: naliubrown@163.com.

Table S1 Clinical characteristics of the PD patients

Variables	Time≤1 month	1<Time≤12	12<Time≤24	24<Time≤36	Time > 36	p-value
Number	16	22	20	16	14	
PD time (months)	1.00(1.00-1.00)	6.00(2.00-8.50)	19.00(18.00-20.75)	28.00(26.00-35.25)	60.50(42.50-77.75)	
Age (years)	62.31±14.63	66.43±11.86	66.53±14.44	57.36±13.43	58.42±14.02	0.2
Male (%)	13(81.25%)	12(54.55%)	12(60.00%)	10(62.50%)	10(71.43%)	0.609
BMI (kg/m^2)	22.38±2.63	23.21±3.00	24.29±2.87	24.37±3.34	23.91±4.67	0.477
Drink (%)	1(6.25%)	0(0.00%)	0(0.00%)	0(0.00%)	1(7.14%)	0.421
Smoke (%)	4(25.0%)	0(0.00%)	3(15.00%)	1(6.25%)	1(7.14%)	0.126
Serum albumin (%)	30.77±5.00	31.3±5.19	32.58±4.73	33.22±5.37	33.67±4.66	0.414
TC (mmol/L)	3.71(2.97-5.17	3.80(3.01-4.34)	3.98(3.57-5.14)	3.54(2.94-4.45)	4.00(3.52-4.62)	0.6
TG (mmol/L)	1.36(0.93-2.08)	1.62(1.05-2.09)	1.79(1.14-2.56)	1.75(1.59-2.70)	2.58(1.34-3.60)	0.579
HDL-C (mmol/L)	1.11(0.69-1.36)	0.95(0.77-1.16)	0.80(0.65-1.18)	0.74(0.71-0.94)	0.82(0.61-1.03)	0.079
LDL-C (mmol/L)	1.89(1.20-2.57)	2.37(1.78-3.08)	2.42(1.77-3.14)	2.29(1.72-2.92)	2.49(2.00-2.95)	0.504
Cr (μmol/L)	648.50(478.22-807.32)	688.20(566.43-896.69)	833.76(605.36-1088.40)	794.84(684.76-1055.78)	1191.00(864.50-1191.00)	0
BUN (mmol/L)	21.69(9.88-28.18)	18.85(14.86-22.80)	18.87(14.13-26.25)	17.46(13.42-20.28)	17.41(15.29-22.88)	0.803
Sodium (mmol/L)	140.28±2.97	140.62±2.85	138.42±3.10	137.81±4.75	141.04±2.37	0.02
Potassium (mmol/L)	4.23±0.75	3.79±0.63	3.85±0.76	3.90±0.50	3.67±0.77	0.225
Calcium (mmol/L)	2.16±0.25	2.25±0.37	2.24±0.24	2.22±0.17	2.25±0，26	0.867
Phosphorus (mmol/L)	1.28±0.42	1.59±0.35	1.63±0.45	1.50±0.49	1.78±0.65	0.054
Hypertension (%)	11(68.75%)	14(63.63%)	9(45.00%)	10(62.50%)	6(42.86%)	0.446
Dyslipidemia (%)	2(12.5%)	5(22.73%)	6(30.00%)	6(27.50%)	5(35.71%)	0.506
Diabetes mellitus (%)	3(18.75%)	8(36.36%)	5(25.00%)	5(31.25%)	1(7.14%)	0.346

Time is measured in months. The continuous variables are reported as means ± SD and categorical variables are presented as percentages. In case of nonparametric data distribution medians with inter quartile range (IQR) are presented. Abbreviations: BMI: body mass index; TC: total cholesterol; TG: triglyceride; HDL-C: high density lipoprotein cholesterol; LDL-C: low density lipoprotein cholesterol; Cr: creatinine; BUN: blood urea nitrogen.
